# Pedal acceleration time is related to arterial stiffness in patients with chronic limb-threatening ischemia

**DOI:** 10.1590/1677-5449.202300492

**Published:** 2025-02-24

**Authors:** Daniel Mendes-Pinto, Larissa Jardim Melo, Guilherme Galvone Fonseca Costa, Izabella Campolina Santos, Ana Paula Pires Silva, Roberto Lucas Senna de Avelar, Guilherme de Castro-Santos, Maria da Glória Rodrigues-Machado

**Affiliations:** 1 Hospital Felício Rocho, Belo Horizonte, MG, Brasil.; 2 Faculdade Ciências Médicas de Minas Gerais, Belo Horizonte, MG, Brasil.; 3 Universidade Federal de Minas Gerais – UFMG, Belo Horizonte, MG, Brasil.

**Keywords:** chronic limb-threatening ischemia, arterial stiffness, Doppler ultrasonography

## Abstract

**Background:**

Pedal acceleration time (PAT) is a novel indicator of peripheral arterial disease in the lower limbs. Elevated PAT values are associated with worse limb ischemia. Arterial stiffness indexes are another class of indicators recently studied in patients with chronic limb-threatening ischemia (CLTI). The correlation between PAT and arterial stiffness has not yet been established.

**Objectives:**

To analyze correlations between PAT and arterial stiffness indexes in patients with CLTI.

**Methods:**

A cross-sectional analysis was conducted of patients with CLTI from August to December of 2022. The PAT measurements were performed using a vascular ultrasound machine and stiffness indexes were measured using a brachial artery oscillometry unit. An analysis was conducted of the correlations between central blood pressure, peripheral blood pressure, arterial stiffness, and PAT.

**Results:**

A total of 55 patients were analyzed, of whom 23 were women and 83.6% had diabetes. Mean PAT was 166.6 ms; mean pulse wave velocity (PWV) was 11.8 m/s, and the mean augmentation index corrected for a heart rate of 75 beats per minute (AIx@75) was 29.8%. There were positive correlations between PAT and PWV (Spearman r = 0.69; p < 0.001) and between PAT and AIx@75 (Spearman r = 0.59, p < 0.001) and an inverse relationship with the ankle/brachial index (Spearman r = -0.79; p < 0.001).

**Conclusions:**

There is a correlation between arterial stiffness indexes and PAT in patients with CLTI. These indicators are important for quantification of limb ischemia.

## INTRODUCTION

The prevalence of peripheral arterial disease (PAD) has increased over recent decades, especially in developing countries.^1^ There are established predictors of PAD progression, such as smoking, diabetes, and chronic renal failure. Advanced degrees of ischemia, infection, and the extent of ischemic lesions in patients with diabetes (graded with the Wound, Ischemia, and Foot Infection [WIfI] classification) are associated with amputations, increased mortality, and increased arterial stiffness.^2,3^ Serological markers such as C-reactive protein and interleukin-6 have been associated with increased mortality in patients with chronic limb-threatening ischemia (CLTI).^4^ Measures of the pressure in the ischemic limb, primarily the ankle/brachial index (ABI), are some of the most widely adopted markers of PAD and CLTI.

The ABI contributes to differential diagnosis of PAD and is related to prognosis. An ABI below 0.9 is associated with a risk ratio of 2.5 for overall mortality and 2.9 for mortality from cardiovascular events.^5^ One limitation of the ABI is calcification of the arteries of the leg, making them incompressible. Measurements of the pressure in the toes, the toe/brachial index, and transcutaneous oxygen pressure are alternative methods for quantifying arterial disease, but they are less widely adopted because of the cost of the equipment used.^6^ Therefore, new markers that could help in management of patients with CLTI are being studied.

Pedal acceleration time (PAT) is a measure of the time elapsed between the start of the systolic curve and peak systole as determined from the Doppler spectral curve for arteries of the foot. It is measured using an ultrasound machine with pulsed Doppler, using a linear transducer and frequencies from 3 to 12 MHz; the same protocol used for duplex scanning of the arteries of the lower limbs.^7^ Pedal acceleration time is measured in the arteries of the foot, such as the retromaleolar posterior tibial artery, the dorsal artery of the foot, the arcuate artery, and the plantar arteries.^7,8^ In the initial study describing the technique, Sommerset et al.^7^ calculated the correlation with the ABI, showing that the more prolonged the PAT, the greater the degree of ischemia. In addition to the inverse correlation with ABI, PAT is also correlated in the same direction with toe pressure and the toe/brachial index.^9^

Arterial stiffness indexes are the markers most recently associated with advanced atherosclerosis and PAD.^10^ Arterial stiffness has been quantified in several different ways, with emphasis on portable equipment, such as the brachial artery oscillometry unit.^11^ In common with pulse wave velocity (PWV), the augmentation index (AIx), and augmentation pressure (AP), increased stiffness indexes are associated with higher mortality due to cardiovascular events and, in patients with CLTI, with increased risk of amputations and death.^12,13^

The objective of this study is to analyze whether there is a correlation between new markers used in PAD: PAT and the arterial stiffness indexes. Our hypothesis is that there is a correlation between these indexes, which would increase the importance of these indicators in assessment of patients with limb ischemia.

## METHODS

This is a cross-sectional analysis of patients seen at a vascular surgery clinic from August 2022 to December 2022. The study was approved by the local Research Ethics Committee, under decision number 5,476,733. Patients aged 18 years or older were recruited if they had CLTI classified as ischemic pain at rest or ischemic trophic ulcers on the lower limbs. Patients with ABI over 1.3 were not included in the study sample. Patients were excluded if they had advanced heart failure classified as New York Heart Association functional class IV, as were expectant mothers. Epidemiological and clinical variables were collected and arterial stiffness, PAT, and ABI measurements were recorded. Patients were defined as smokers if they were smoking at the time of the interview or had quit within the previous 6 months.

Each patient’s PAT was measured with the linear ultrasound transducer placed over the arteries of the ischemic foot: the dorsalis pedis artery, posterior tibial artery, and plantar arteries. Measuring PAT with vascular ultrasound takes approximately 5 minutes.

Arterial stiffness indexes were measured using Mobil-O-Graph (IEM, Stolberg, Germany) arterial oscillometry equipment (Figure S1, Supplementary Material). This is a portable device consisting of a control unit connected to a cuff with sensors that transfer the oscillometric measurement information to a portable computer.^14^ Using mathematical protocols involving patient age, sex, height, and weight, it is possible to infer arterial stiffness and central blood pressure measurements in approximately 15 minutes. The following pressure measurements were recorded: central systolic blood pressure (SBPc), central diastolic blood pressure (DBPc), central pulse pressure (PPc), peripheral systolic blood pressure (SBPp), peripheral diastolic blood pressure (DBPp), peripheral pulse pressure (PPp); and arterial stiffness indexes: PWV, augmentation index corrected for a heart rate of 75 beats per minute (AIx@75), and AP.

The ABI was measured for both lower limbs with the patient in a supine position, using a portable continuous wave Doppler ultrasound machine with a 7.5 mHz probe (Microem, Ribeirão Preto, Brazil). Systolic pressures were measured at the dorsalis pedis and posterior tibial arteries, bilaterally, using the Doppler machine and an aneroid sphygmomanometer on the leg. Only the more ischemic limb was considered for analysis, determined by the presence of trophic ulcers that threaten viability.

An analysis was conducted of the correlations between PAT, pressure measurements, and arterial stiffness indexes. The normality of data was determined with the Shapiro test. Spearman nonparametric correlation coefficients were calculated, where the closer to 1 the result, the stronger the correlation. Our objective in testing for correlations between variables was to achieve a significant result (p < 0.05) with 80% test power to detect correlation coefficients of at least 0.4. The minimum sample size for these parameters was 46 participants.^15^ The study was planned with the aid of the STROBE recommendations for cross-sectional studies.^16^ We used the statistical software Graphpad Prism version 9 for analyses of normality and correlations and to plot graphs.

## RESULTS

Data were analyzed from 55 patients, 23 women (41.8%), with mean age of 74.5 years (Table 1**)** (Figure S2, Supplementary Material). The majority were diabetes patients (83.6%) and had ischemic lesions classified as Rutherford stage 5 (70.9%). The mean PAT for the sample was 166.6 ms. Peripheral pressures were higher than central pressures, as inferred by the brachial oscillometry unit’s algorithm. The results of arterial stiffness assessments were mean PWV of 11.8 ms, AP of 18.9 mmHg, and AIx@75 of 29.8%.

**Table 1:** Clinical data, central blood pressure, peripheral blood pressure, and arterial stiffness indexes for 55 patients with obstructive arterial disease of the lower limbs.

Variable studied	Value
Age (years)	74.5 (72; 76.9)
Female	23 (41.8%)
Diabetes	46 (83.6%)
Ankle/brachial index – ABI	0.55 (0.51; 0.59)
Rutherford stage 4	8 (3.6%)
Rutherford stage 5	39 (70.9%)
Rutherford stage 6	8 (14.5%)
Pedal acceleration time (ms)	166.6 (152.8 180.3)
Peripheral pressures	
Peripheral systolic blood pressure – SBPp (mmHg)	146.4 (141; 151.8)
Peripheral diastolic blood pressure – DBPp (mmHg)	80.9 (77.7; 84)
Peripheral pulse pressure– PPp (mmHg)	65.5 (61.9; 69)
Central pressures	
Central systolic blood pressure – SBPc (mmHg)	128.9 (124.2; 133.7)
Central diastolic blood pressure - DBPc (mmHg)	82.5 (79.2; 85.7)
Central pulse pressure – PPc (mmHg)	46.4 (43.6; 49.2)
Arterial stiffness indexes	
Pulse wave velocity – PWV (m/s)	11.8 (11.3; 12.4)
Augmentation pressure – AP (mmHg)	18.9 (16.2; 21.5)
Augmentation index – AIx@75 (%)	29.8 (26.8; 32.8)

Data for continuous variables expressed as mean (95% confidence interval).

**Figure 1:**
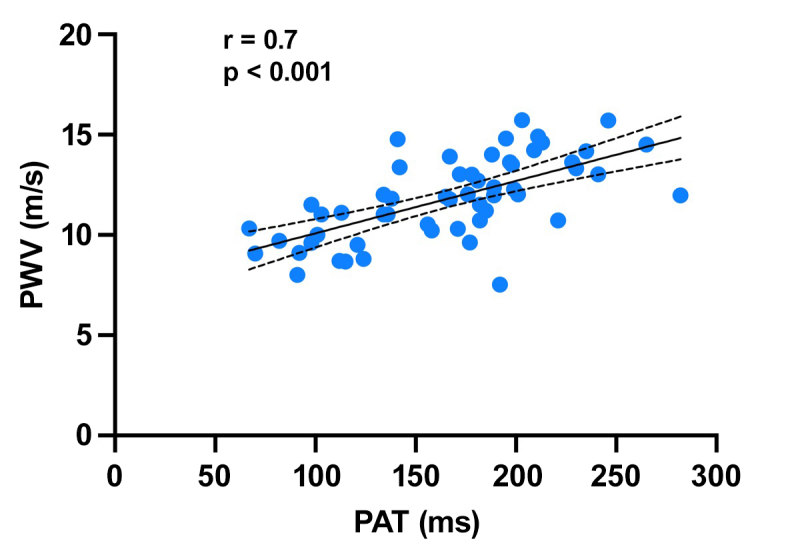
Correlation between PAT (pedal acceleration time) and PWV (pulse wave velocity). r: Spearman correlation coefficient.

**Figure 2:**
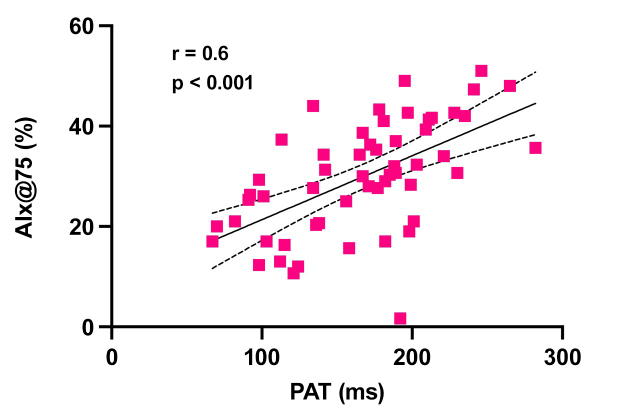
Correlation between PAT (pedal acceleration time) and AIx@75 (augmentation index corrected for a heart rate of 75 beats per minute). r: Spearman correlation coefficient.

**Figure 3:**
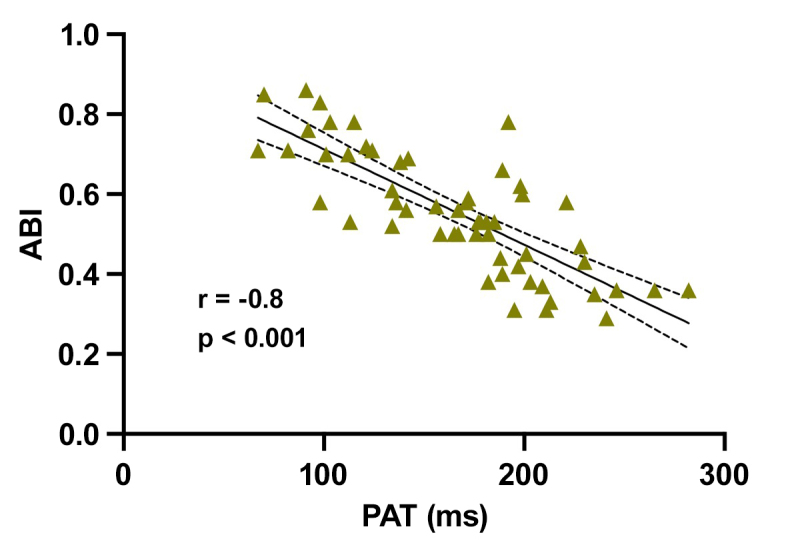
Correlation between PAT (pedal acceleration time) and ABI (ankle/brachial index). r: Spearman correlation coefficient.

Table 2 shows the results of the correlation analyses. There was a statistically significant correlation between PAT and the arterial stiffness indexes analyzed. There was a strong correlation between PAT and PWV (r = 0.69, p < 0.001) (Figure 1); while the correlations with AIx@75 (r = 0.59, p < 0.001) (Figure 2) and AP (r = 0.56, p < 0.001) were weak. There was a strong inverse relationship between PAT and ABI (r = -0.79; p < 0.001) (Figure 3). Correlations between PAT and systolic and diastolic pressures were not significant. However, the correlations between PAT and PPp (r = 0.34, p = 0.01) and PAT and PPc (r = 0.32, p = 0.01) were statistically significant, although weak.

**Table 2:** Analysis of correlations between pedal acceleration time and clinical data and arterial stiffness indexes.

Correlation	r (Spearman)	95% confidence interval	p
PAT vs. age	-0.13	-0.39; 0.15	0.34
PAT vs. PWV	0.69	0.51; 0.81	< 0.001
PAT vs. AIx@75	0.59	0.38; 0.75	< 0.001
PAT vs. AP	0.56	0.34; 0.72	< 0.001
PAT vs. ABI	-0.79	-0.88; -0.67	< 0.001
PAT vs. SBPp	0.21	-0.06; 0.46	0.11
PAT vs. DBPp	-0.06	-0.33; 0.21	0.65
PAT vs. PPp	0.34	0.07; 0.56	0.01
PAT vs. SBPc	0.16	-0.11; 0.42	0.23
PAT vs. DBPc	-0.06	-0.33; 0.20	0.62
PAT vs. PPc	0.32	0.05; 0.54	0.01

PAT = pedal acceleration time; PWV = pulse wave velocity; AIx@75 = augmentation index corrected for a heart rate of 75 beats per minute; AP = augmentation pressure; ABI = ankle/brachial index; SBPp = peripheral systolic blood pressure; DBPp = peripheral diastolic blood pressure; PPp = peripheral pulse pressure; SBPc = central systolic blood pressure; DBPc = central diastolic blood pressure; PPc = central pulse pressure.

## DISCUSSION

Pedal acceleration time is a new marker of PAD that quantifies the degree of limb ischemia.^7,17^ In addition to quantification of PAD, there are also correlations between elevated PAT values and outcomes of ischemia such as major amputations.^8,18,19^ The findings of this study show that there is a strong correlation between PAT and the arterial stiffness indexes, which are also variables that are associated with outcomes of lower limb ischemia such as amputation or death.^13^

The more advanced the degree of limb ischemia, the more prolonged PAT will be.^20^ Moreover, Castro-Santos et al.^8^ have shown that PAT can diagnose limb ischemia stage with sensitivity of at least 80%, when analyzed by the WIfI classification. In order to measure PAT, the Doppler curve is analyzed for arteries of the foot, obtained using vascular ultrasound, which is a technique that is widely used by vascular surgeons and in related specialties.^21,22^ As such, PAT is an indicator of arterial disease that is accessible to a large proportion of patients, since it is obtained with equipment that is used routinely.

Elevated PWV has been associated with cardiovascular complications such as heart attack, stroke, renal failure, and limb-threatening ischemia.^23^ Elevated arterial stiffness levels are associated with injury to target organs via diverse mechanisms, such as microcirculation injury and transmission of pressure directly to small caliber arterioles without the damping effect of arterial elasticity.^24^ Arterial stiffness is associated with left ventricle hypertrophy, heart failure, and renal failure.^25^ Transmission of the pulse wave contributes to cerebral microbleeds which, over the long term, lead to cognitive deficit and dementia.^26^ The data from this study show that PAT is an indicator of systemic arterial disease characterized by increased arterial stiffness indexes.

Advanced arterial stiffness is related to worse outcomes after endovascular or surgical lower limb revascularization.^27^ In one prospective study of patients with CLTI, a PWV exceeding 12.7 m/s was associated with a four times greater risk of amputation or death and an AP exceeding 23 mmHg was associated with a 13 times greater risk.^13^ Moreover, a 10% increase in AIx was related to a 1.8 times increase in the likelihood of occurrence of cardiovascular events and death.^28^ Changes in arterial stiffness affect both adults and children and a cross-sectional study showed that children with obstructive sleep-disordered breathing had elevated AIx@75.^29^ These data show the importance of early diagnosis of arterial stiffness in patients with advanced atherosclerotic disease. As such, the correlation between stiffness indexes and PAT shown in this study contributes to a complete analysis of patients with CLTI, to better manage cases and predict events.

Our data reveal a strong inverse correlation between PAT and ABI. This correlation is compatible with data published by Castro-Santos et al.,^8^ who showed the accuracy of PAT for detecting an ABI < 0.8 in 85% of patients without diabetes and accuracy of 91% in those with diabetes. This is an interesting fact, since presence of calcification of the arteries of the foot is more common in those with diabetes than those without, causing incompressibility of the arteries and an inability to use ABI to quantify arterial disease. In these patients, use of measurement of hallux pressure and the toe/brachial index are options for quantification of perfusion in the foot, but use of the equipment needed is limited by the high cost.^6^ In this respect, analysis of the acceleration time in arteries of the foot using widely-available duplex scan equipment has the potential for widespread use in patients with PAD.

Interestingly, despite the positive correlations with PWV, AIx@75, and AP, there was no correlation between PAT and systolic or diastolic blood pressures, whether peripheral or central. Systolic blood pressures constitute one of the main components associated with arterial stiffness. Both elevated PPp and elevated PPc are associated with increased risk of coronary disease.^27^ Elevated PPc is also associated with increased complications in patients with CLTI who undergo angioplasty.^30^ However, there was a weak correlation with pulse pressures, which show the difference between systolic and diastolic pressures. It is possible that, with the advance of limb ischemia that is reflected by elevated PAT, systolic blood pressure increases without a corresponding reduction in diastolic blood pressure, resulting in the increase in pulse pressure. Elevated pulse pressure is associated with elevated blood pressure^24^ and is possibly a more sensitive marker related to the degree of limb ischemia.

One limitation of this study is the fact that the analysis was cross-sectional, which precludes establishment of relationships of cause and effect between PAT, arterial stiffness indexes, and the outcomes of CLTI. An analysis of PAT and quantifiers of arterial disease, such as the WIfI classification, would be one possibility for analyzing the relationship with degree of ischemia. The fact that a majority of the patients in the sample had diabetes could be considered a confounding factor, since these patients tend to have higher pressures in their feet because of arterial calcification. However, this possibility was minimized by the sample selection strategy excluding people with ABI exceeding 1.3. Additionally, medications that affect oscillometric analysis of the brachial artery, such as antihypertensives, were not withdrawn for measurement of arterial stiffness and neither were dietary interventions implemented to exclude potential confounders such as caffeine. Finally, the data analyzed were from a single center, which reduces the external validity of the findings.

The correlation between arterial stiffness indexes and PAT observed in this study confirms the value of this indicator as data that it is important to determine in patients with CLTI. This study indicates that the practice of measuring PAT could contribute to better classification of patients with CLTI by specialists. Prospective studies could better analyze the predictive value of PAT in patients with elevated arterial stiffness with regard to outcomes of limb ischemia.

## Supplementary Material

Supplementary material accompanies this paper.

Figure S1. Patient with the brachial oscillometry unit measuring arterial stiffness (permission for photo granted).

Figure S2. Flow diagram.

This material is available as part of the online article from https://doi.org/10.1590/1677-5449.202300492
