# Supplementary figures and images for: Pedal acceleration time is related to arterial stiffness in patients with chronic limb-threatening ischemia
Source: J Vasc Bras. 2025 Feb 24;24:e20230049. doi: 10.1590/1677-5449.202300492 (PMC11895777; doi:10.1590/1677-5449.202300492)

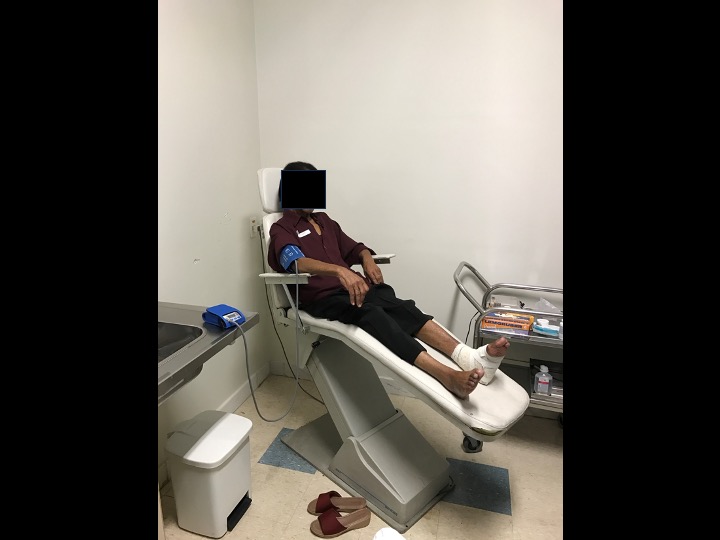

Supplement: Figure S1. [file jvb-24-e20230049-g04-en.tif]

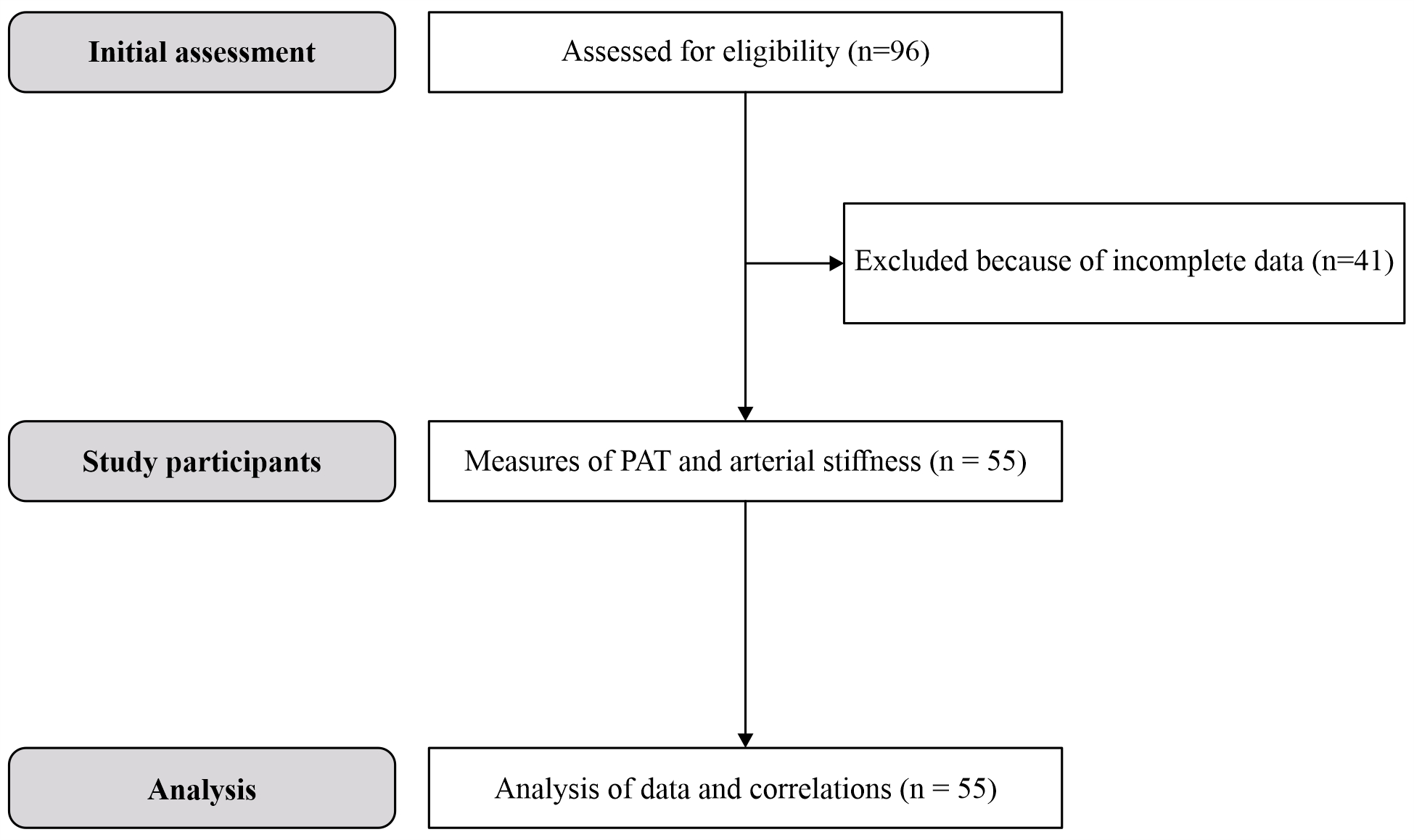

Supplement: Figure S2. [file jvb-24-e20230049-g05-en.tif]

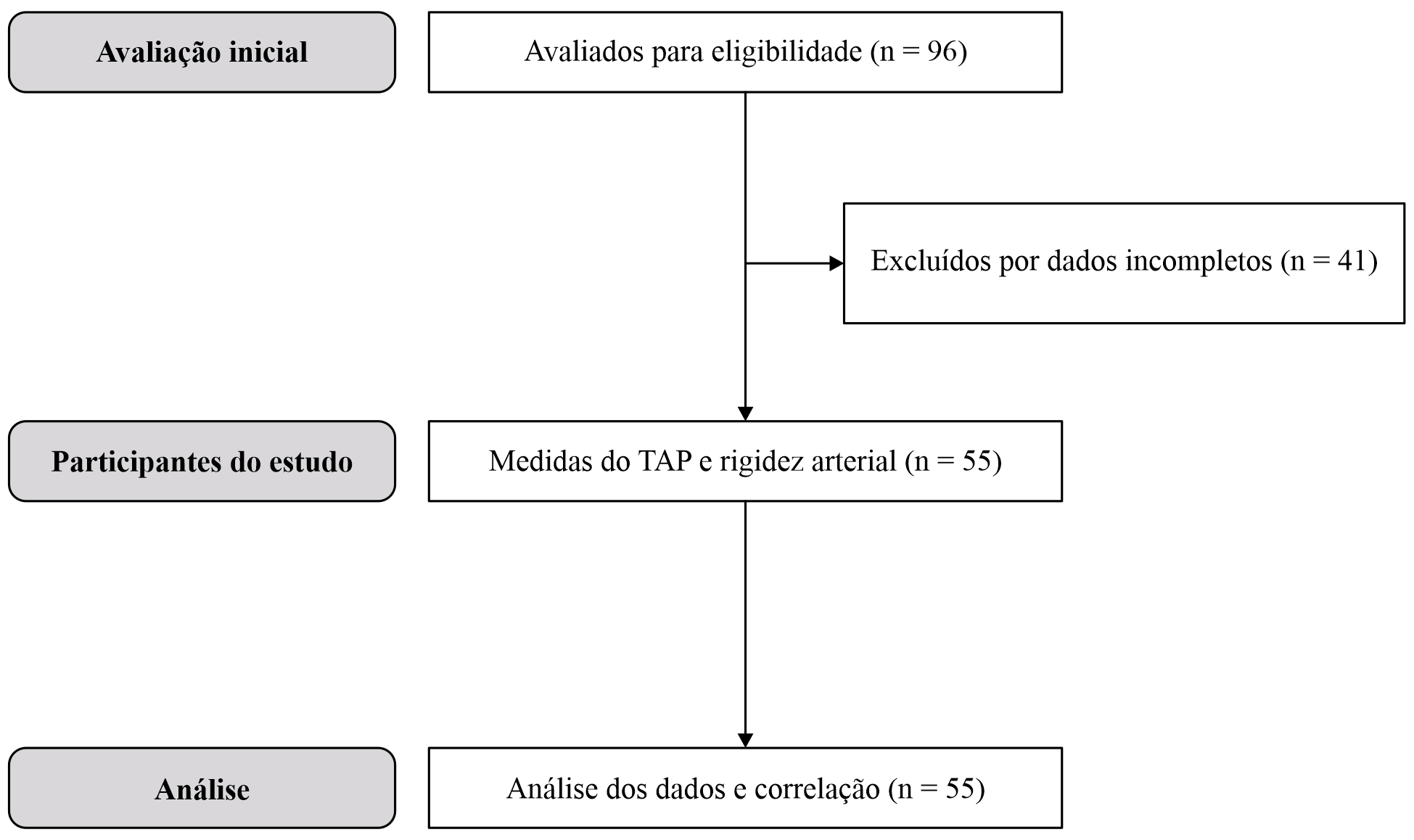

Supplement: Figura S2. [file jvb-24-e20230049-g05.tif]
