# Supplementary material for: Pedal acceleration time is related to arterial stiffness in patients with chronic limb-threatening ischemia
Source: J Vasc Bras. 2025 Feb 24;24:e20230049. doi: 10.1590/1677-5449.202300492 (PMC11895777; doi:10.1590/1677-5449.202300492)
Supplement: Translation

# Translation: Tempo de aceleração plantar está relacionado a rigidez arterial em pacientes com isquemia crônica ameaçadora ao membro

**DOI:** 10.1590/1677-5449.202300491

**Authors:** Daniel Mendes-Pinto, Larissa Jardim Melo, Guilherme Galvone Fonseca Costa, Izabella Campolina Santos, Ana Paula Pires Silva, Roberto Lucas Senna de Avelar, Guilherme de Castro-Santos, Maria da Glória Rodrigues-Machado

**Affiliations:** 1 Hospital Felício Rocho, Belo Horizonte, MG, Brasil.; 2 Faculdade Ciências Médicas de Minas Gerais, Belo Horizonte, MG, Brasil.; 3 Universidade Federal de Minas Gerais – UFMG, Belo Horizonte, MG, Brasil.

**Keywords:** isquemia crônica crítica de membro, rigidez vascular, ultrassonografia Doppler

## Abstract

**Contexto:**

O tempo de aceleração plantar (TAP) é um novo indicador da doença arterial periférica de membros inferiores. Valores elevados do TAP estão associados a pior isquemia do membro. Os índices de rigidez arterial são também indicadores recentemente estudados em pacientes com isquemia crônica ameaçadora ao membro (*chronic limb-threatening ischemia,* CLTI). A correlação entre o TAP e a rigidez arterial ainda não foi estabelecida.

**Objetivos:**

Analisar a correlação entre TAP e índices de rigidez arterial em pacientes com CLTI.

**Métodos:**

Foi realizada a análise transversal de pacientes com CLTI entre agosto e dezembro de 2022. A medida do TAP foi feita utilizando aparelho de ultrassom vascular, e a medida dos índices de rigidez, utilizando aparelho de oscilometria da artéria braquial. Foi feita uma análise de correlação entre índices de pressão arterial central, pressão arterial periférica, de rigidez arterial e o TAP.

**Resultados:**

Foram analisados 55 pacientes, sendo 23 mulheres e 83,6% diabéticos. O TAP médio foi de 166,6 ms; a velocidade de onda de pulso (VOP) média foi de 11,8 m/s; e o índice de aumento médio corrigido para frequência cardíaca de 75 batimentos por minuto (AIx@75) foi de 29,8%. Houve correlação positiva entre o TAP e a VOP (Spearman = 0,69; p < 0,001), entre o TAP e o AI@75 (Spearman = 0,59, p < 0,001) e relação inversa com o índice tornozelo/braço (Spearman = -0,79; p < 0,001).

**Conclusões:**

Há correlação entre índices de rigidez arterial e o TAP em pacientes com CLTI. Esses indicadores são de importância na quantificação da isquemia do membro.

## INTRODUÇÃO

A prevalência da doença arterial periférica (DAP) tem aumentado nas últimas décadas, em especial em países em desenvolvimento^1^ . Há preditores estabelecidos para a progressão da DAP, como o tabagismo, a diabetes e a insuficiência renal crônica. Graus avançados de isquemia, infecção e extensão de lesões isquêmicas em diabéticos, graduados pela classificação *Wound, Ischemia and Foot Infection* (WIfI), estão associados a amputações, mortalidade aumentada e aumento da rigidez das artérias^2,3^ . Marcadores sorológicos como a proteína C reativa e interleucina-6 têm sido associados a mortalidade aumentada em pacientes com isquemia crônica ameaçadora ao membro (*chronic limb threatening ischemia –* CLTI)^4^ . As medidas de pressão do membro isquêmico, principalmente o índice tornozelo/braço (ITB) estão entre os marcadores da DAP e CLTI mais difundidos.

O ITB contribui para o diagnóstico diferencial da DAP e está relacionado com o prognóstico. O ITB abaixo de 0,9 está associado a uma razão de risco de 2,5 para mortalidade geral e de 2,9 para mortalidade devido a eventos cardiovasculares^5^ . Uma limitação do ITB é a calcificação das artérias da perna, que as torna incompressíveis. A medida de pressão em dedos do pé, o índice dedo/braço e a pressão transcutânea de oxigênio são medidas alternativas para quantificar a doença arterial, porém, são métodos menos difundidos devido ao custo dos equipamentos utilizados^6^ . Assim, têm sido estudados novos marcadores que possam auxiliar na condução dos pacientes com CLTI.

O tempo de aceleração pedal (TAP) consiste na medida de tempo entre o início da curva sistólica e o pico da sístole feita na curva de Doppler espectral em artérias do pé. A medida é feita com aparelho de ultrassom com Doppler pulsado, com tansdutor linear e frequências entre 3 e 12 MHz, o mesmo protocolo usado para o *duplex-scan* das artérias em membros inferiores^7^ . A medida do TAP é feita em artérias do pé, como a artéria tibial posterior retromaleolar, artéria dorsal do pé, artéria arqueada e artérias plantares^7,8^ . No estudo inicial que descreve a técnica, Sommerset et al.^7^ fazem uma correlação com o ITB de modo que, quanto mais prolongado o TAP, maior o grau de isquemia. Além da correlação inversa com o ITB, o TAP correlaciona-se da mesma forma com a pressão de dedo e índice dedo/braço^9^ .

Os índices de rigidez arterial são os marcadores mais recentes associados a aterosclerose avançada e DAP^10^ . A quantificação da rigidez arterial tem sido feita de várias maneiras, com destaque para aparelhos portáteis como o de oscilometria da artéria braquial^11^ . O aumento de índices de rigidez como a velocidade da onda de pulso (VOP), o índice de aumento (*augmentation index –* AIx) e a pressão de aumento (*augmentation pressure* – AP) estão associados ao aumento da mortalidade por eventos cardiovasculares e, em pacientes com CLTI, ao aumento do risco de amputações e óbito^12,13^ .

O objetivo deste estudo é analisar se há correlação entre novos marcadores utilizados na DAP: o TAP e os índices de rigidez arterial. Nossa hipótese é que há correlação entre esses índices, o que aumentaria a importância desses indicadores na avaliação dos pacientes com isquemia de membro.

## MÉTODOS

Esta é uma análise transversal de pacientes atendidos no ambulatório de cirurgia vascular entre agosto de 2022 e dezembro de 2022. O estudo foi aprovado pelo Comitê de Ética em Pesquisa local, sob o parecer número 5.476.733. Foram incluídos pacientes com idade maior ou igual a 18 anos e que apresentavam CLTI classificados como dor isquêmica em repouso ou lesões tróficas isquêmicas em membros inferiores. Pacientes com ITB acima de 1,3 não foram incluídos no estudo. Foram excluídos pacientes com insuficiência cardíaca avançada classificados como classe funcional IV da *New York Heart Association* e gestantes. Foram coletadas variáveis epidemiológicas, clínicas, medidas de rigidez arterial, do TAP e do ITB. Foram definidos como tabagistas aqueles que fumavam à época da entrevista ou que haviam interrompido o hábito nos últimos seis meses.

O TAP foi medido posicionando-se o transdutor linear do ultrassom sob as artérias do pé isquêmico: artéria pediosa, artéria tibial posterior e plantares. A medida do TAP com ultrassom vascular dura aproximadamente 5 minutos.

A medida dos índices de rigidez arterial foi feita com o aparelho Mobil-O-Graph (IEM, Stolberg, Alemanha) de oscilometria arterial (Figura S1, Material Suplementar). Trata-se de um aparelho portátil que consiste em uma unidade controladora conectada a um manguito com sensores que transferem a informação das medidas oscilométricas para um computador portátil^14^ . Por meio de protocolos matemáticos incluindo idade, sexo, altura e peso do paciente, é possível inferir as medidas de rigidez arterial e pressão arterial central em um período de aproximadamente 15 minutos. Foram coletadas medidas de pressão: pressão arterial sistólica central (PASc), pressão arterial diastólica central (PADc), pressão de pulso central (PPc), pressão arterial sistólica periférica (PASp), pressão arterial diastólica periférica (PADp), pressão de pulso periférica (PPp); índices de rigidez arterial: VOP, índice aumento corrigido para a frequência cardíaca de 75 batimentos por minuto (AIx@75) e AP.

O ITB foi medido em ambos os membros inferiores com o paciente em posição supina, com uso de aparelho de Doppler de ondas contínuas portátil com sonda de 7,5 mHz (Microem, Ribeirão Preto, Brasil). Foram medidas as pressões sistólicas nas artérias pediosa e tibial posterior, bilateralmente, com uso do Doppler e esfigmomanômetro aneroide na perna. Somente o membro mais isquêmico foi considerado para a análise, caracterizado pela presença de lesões tróficas ameaçadoras à viabilidade.

Foi feita a análise de correlação entre o TAP e as medidas de pressão e os índices de rigidez arterial. A normalidade dos dados foi feita pelo teste de Shappiro. O índice de correlação não paramétrico de Spearman foi calculado, sendo que, quanto mais próximo a 1, mais forte a correlação. Nosso objetivo na correlação entre variáveis foi chegar a um resultado significativo (p < 0,05) com poder do teste de 80% para detectar um coeficiente de correlação de pelo menos 0,4; a amostra mínima para esses parâmetros foi de 46 participantes^15^ . O estudo foi planejado com o auxílio das orientações STROBE para estudos transversais^16^ . Utilizamos o software estatístico *Graphpad Prism* versão 9 para as análises de normalidade, de correlação e gráficos.

## RESULTADOS

Foram analisados dados de 55 pacientes, 23 mulheres (41,8%), média de idade 74,5 anos (Tabela 1) (Figura S2, Material Suplementar). A maioria era de diabéticos (83,6%) e com lesões isquêmicas classificadas como Rutherford 5 (70,9%). O TAP médio da amostra foi de 166,6 ms. As pressões periféricas foram maiores que as pressões centrais, inferidas pelo algoritmo do aparelho de oscilometria braquial. Os índices de rigidez arterial avaliados foram a VOP média de 11,8 ms, AP de 18,9 mmHg e AIx@75 de 29,8%.

**Tabela 1:** Dados clínicos, de pressão arterial central, periférica e de rigidez arterial de 55 pacientes com doença arterial obstrutiva em membros inferiores.

Variável estudada	Valor
Idade (anos)	74,5 (72; 76,9)
Sexo feminino	23 (41,8%)
Diabéticos	46 (83,6%)
Índice tornozelo/braço – ITB	0,55 (0,51; 0,59)
Rutherford 4	8 (3,6%)
Rutherford 5	39 (70,9%)
Rutherford 6	8 (14,5%)
Tempo de aceleração plantar (ms)	166,6 (152,8 180,3)
Pressões periféricas	
Pressão arterial sistólica periférica – PASp (mmHg)	146,4 (141; 151,8)
Pressão arterial diastólica periférica – PADp (mmHg)	80,9 (77,7; 84)
Pressão de pulso periférica – PPp (mmHg)	65,5 (61,9; 69)
Pressões centrais	
Pressão arterial sistólica central – PASc (mmHg)	128,9 (124,2; 133,7)
Pressão arterial diastólica central - PADc (mmHg)	82,5 (79,2; 85,7)
Pressão de pulso central – PPc (mmHg)	46,4 (43,6; 49,2)
Índices de rigidez arterial	
Velocidade da onda de pulso – VOP (m/s)	11,8 (11,3; 12,4)
Pressão de aumento – AP (mmHg)	18,9 (16,2; 21,5)
Índice de aumento – AIx@75 (%)	29,8 (26,8; 32,8)

Dados de variáveis contínuas apresentados como média (intervalo de confiança 95%).

As análises de correlação são mostradas na Tabela 2. Houve correlação estatisticamente significativa entre o TAP e os índices de rigidez arterial analisados. A correlação do TAP com a VOP foi forte (r = 0,69, p < 0,001) (Figura 1); as correlações com o AIx@75 (r = 0,59, p < 0,001) (Figura 2) e AP (r = 0,56, p < 0,001) foram moderadas. Houve forte relação inversa entre o TAP e o ITB (r = -0,79; p < 0,001) (Figura 3). As correlações do TAP com pressões sistólicas e diastólicas não foram significativas. No entanto, a correlação do TAP com a PPp (r = 0,34, p = 0,01) e PPc (r = 0,32, p = 0,01), apesar de fracas, foram estatisticamente significativas.

**Tabela 2:** Análise de correlação entre o tempo de aceleração plantar e dados clínicos e de rigidez arterial.

Correlação	r (Spearman)	Intervalo de confiança 95%	Valor-p
TAP vs. idade	-0,13	-0,39; 0,15	0,34
TAP vs. VOP	0,69	0,51; 0,81	< 0,001
TAP vs. AIx@75	0,59	0,38; 0,75	< 0,001
TAP vs. AP	0,56	0,34; 0,72	< 0,001
TAP vs. ITB	-0,79	-0,88; -0,67	< 0,001
TAP vs. PASp	0,21	-0,06; 0,46	0,11
TAP vs. PADp	-0,06	-0,33; 0,21	0,65
TAP vs. PPp	0,34	0,07; 0,56	0,01
TAP vs. PASc	0,16	-0,11; 0,42	0,23
TAP vs. PADc	-0,06	-0,33; 0,20	0,62
TAP vs. PPc	0,32	0,05; 0,54	0,01

TAP = tempo de aceleração plantar; VOP = velocidade da onda de pulso; AIx@75 = índice de aumento corrigido para frequência cardíaca de 75 batimentos por minuto; AP = pressão de aumento; ITB = índice tornozelo/braço; PASp = pressão arterial sistólica periférica; PADp = pressão arterial diastólica periférica; PPp = pressão de pulso periférica; PASc = pressão arterial sistólica central; PADc = pressão arterial diastólica central; PPc = pressão de pulso central.

**Figura 1:** Correlação entre TAP (tempo de aceleração plantar) e VOP (velocidade de onda de pulso). r: coeficiente de correlação de Spearman.

**Figura 2:** Correlação entre TAP (tempo de aceleração plantar) e AIx@75 (índice de aumento corrigido para frequência cardíaca de 75 batimentos por minuto). r: coeficiente de correlação de Spearman.

**Figura 3:** Correlação entre TAP (tempo de aceleração plantar) e ITB (índice tornozelo/braço). r: coeficiente de correlação de Spearman.

## DISCUSSÃO

O TAP é um novo marcador da DAP que quantifica o grau de isquemia do membro^7,17^ . Além da quantificação da DAP, há correlação entre valores elevados do TAP e desfechos da isquemia como amputações maiores^8,18,19^ . Os achados deste estudo mostram que há forte correlação entre o TAP e os índices de rigidez arterial, que são variáveis também associadas a desfechos da isquemia de membro inferior como a amputação ou o óbito^13^ .

Quanto mais avançado o grau de isquemia do membro, mais prolongado é o TAP^20^ . Além disso, Castro-Santos et al.^8^ mostraram que o TAP pode diagnosticar os graus de isquemia do membro com sensibilidade de pelo menos 80%, quando analisados pela classificação WIfI. Para a medida do TAP, são feitas análises da curva Doppler em artérias do pé por meio do ultrassom vascular, um meio que tem sido amplamente utilizado entre os cirurgiões vasculares e especialidades afins^21,22^ . Desse modo, o TAP é um indicador de doença arterial acessível a um grande número de pacientes uma vez que sua obtenção exige equipamentos de uso rotineiro.

A VOP elevada está associada a complicações cardiovasculares como o infarto, o acidente vascular cerebral, a insuficiência renal e a isquemia ameaçadora a membros^23^ . Níveis elevados de rigidez arterial estão associados a lesões de órgãos-alvo por mecanismos diversos, como a lesão da microcirculação e a transmissão da pressão diretamente para arteríolas de pequeno calibre sem o efeito de amortecimento da elasticidade arterial^24^ . A rigidez arterial está associada a hipertrofia do ventrículo esquerdo, insuficiência cardíaca e insuficiência renal^25^ . A transmissão da onda de pulso contribui para microinfartos cerebrais que, a longo prazo, levam a déficit cognitivo e demência^26^ . Os dados deste estudo mostram que o TAP é um indicador da doença arterial sistêmica caracterizada pelos índices aumentados de rigidez arterial.

A rigidez arterial avançada está relacionada a resultados piores da revascularização endovascular ou cirúrgica do membro inferior^27^ . Em um estudo prospectivo em pacientes com CLTI, a VOP acima de 12,7 m/s foi associada a um risco quatro vezes maior de amputação ou óbito, e a AP acima de 23 mmHg, a um risco 13 vezes maior^13^ . Além disso, o aumento do AIx em 10% foi relacionado a um aumento da chance em 1,8 de ocorrência de eventos cardiovasculares e óbito^28^ . As alterações de rigidez arterial afetam tanto adultos quanto crianças; um estudo transversal mostrou que crianças com distúrbio respiratório obstrutivo do sono apresentam níveis elevados de AIx@75^29^ . Tais dados mostram a importância do diagnóstico precoce da rigidez arterial em pacientes com doença aterosclerótica avançada. Nesse sentido, a correlação entre os índices de rigidez e o TAP mostrada neste estudo vem ao encontro à análise completa do paciente com CLTI, para melhor condução do caso e predição dos eventos.

Nossos dados mostram a forte correlação inversa entre o TAP e o ITB. Tal correlação é condizente com os dados de Castro-Santos et al.^8^ , que mostraram uma acurácia do TAP em detectar o ITB < 0,8 em 85% em não diabéticos e uma acurácia de 91% em diabéticos. Tal fato é interessante, uma vez que, em diabéticos, a presença de calcificação das artérias do pé é mais comum que em não diabéticos, levando à incompressibilidade das artérias e à incapacidade de utilização do ITB para quantificar a doença arterial. Nesses pacientes, a utilização de medidas de pressão do hálux e o índice dedo/braço são opções para a quantificação da perfusão no pé, porém, o uso desses aparelhos é limitado devido ao alto custo^6^ . Nesse sentido, a análise do tempo de aceleração em artérias do pé utilizando aparelhos de *duplex-scan* que são amplamente difundidos tem potencial para ser utilizada amplamente nos pacientes com DAP.

Interessantemente, apesar da correlação positiva com a VOP, AIx@75 e AP, não houve correlação do TAP com as pressões arterial sistólica e diastólica, sejam periféricas ou centrais. As pressões arteriais sistólicas são um dos principais componentes associados à rigidez arterial. Tanto a PPp quanto a PPc elevadas estão associadas ao risco de aumento de doença coronariana^27^ . A PPc elevada também está associada ao aumento de complicações em pacientes com CLTI submetidos a angioplastia^30^ . No entanto, houve uma fraca correlação com as pressões de pulso, que mostram a diferença entre as pressões arteriais sistólicas e diastólicas. É possível que, com o avançar da isquemia do membro refletido pelo TAP elevado, ocorra aumento da pressão arterial sistólica, sem redução correspondente da pressão arterial diastólica, resultando no aumento da pressão de pulso. A pressão de pulso elevada está associada a níveis elevados de pressão arterial^24^ e, talvez, seja um marcador mais sensível relacionado ao grau de isquemia do membro.

Uma limitação deste estudo é o fato de ser uma análise transversal, o que impede o estabelecimento de relações de causa e efeito entre o TAP, os índices de rigidez arterial e os desfechos da CLTI. Uma análise entre o TAP e quantificadores da doença arterial, como a classificação WIfI, seria uma possibilidade para analisar a relação com o grau de isquemia. O fato de diabéticos serem a maioria na amostra pode ser considerado um fator de confundimento, uma vez que esses pacientes tendem a ter índices pressóricos mais altos nos pés devido à calcificação arterial; no entanto, isso foi minimizado pelo fato de a amostra não incluir pessoas com ITB acima de 1,3. Além disso, as medicações que influenciam a análise oscilométrica da artéria braquial, como os hipotensores, não foram suspensas para a medida de rigidez arterial; do mesmo modo, não foram feitas intervenções dietéticas para suspender potenciais confundidores como a cafeína. Por fim, os dados foram analisados em um único centro, o que reduz a validade externa dos achados.

A correlação entre os índices de rigidez arterial e o TAP observada neste estudo corrobora o valor deste como um dado importante a ser obtido em pacientes com CLTI. Este estudo acrescenta que a prática de medida do TAP pode contribuir para a melhor classificação do paciente com CLTI feita por especialistas. Estudos prospectivos poderão analisar melhor o efeito preditor do TAP em pacientes com rigidez arterial elevada em relação aos desfechos da isquemia de membro.

## Material Suplementar

Este artigo acompanha material suplementar.

Figura S1. Paciente com aparelho de oscilometria braquial para medida de rigidez arterial (foto com permissão).

Figura S2. Diagrama de fluxo.

Este material está disponível como parte da versão online do artigo na página https://doi.org/10.1590/1677-5449.202300491
